# LOX family and ZFPM2 as novel diagnostic biomarkers for malignant pleural mesothelioma

**DOI:** 10.1186/s40364-019-0180-0

**Published:** 2020-01-08

**Authors:** Min-Kyu Kim, Hyun-won Kim, Mirae Jang, Sung Soo Oh, Suk-Joong Yong, Yangsik Jeong, Soon-Hee Jung, Jong-Whan Choi

**Affiliations:** 1 Departments of Biochemistry, Yonsei University Wonju College of Medicine, Wonju, Gangwon-do Republic of Korea; 2 Departments of Global Medical Science, Yonsei University Wonju College of Medicine, Wonju, Gangwon-do Republic of Korea; 3 Departments of Institute of Lifestyle Medicine, Yonsei University Wonju College of Medicine, Wonju, Gangwon-do Republic of Korea; 4 Departments of Pathology, Yonsei University Wonju College of Medicine, Wonju, Gangwon-do Republic of Korea; 5 Departments of Preventive Medicine, Yonsei University Wonju College of Medicine, Wonju, Gangwon-do Republic of Korea; 6 Departments of Internal Medicine, Yonsei University Wonju College of Medicine, Wonju, Gangwon-do Republic of Korea

**Keywords:** LOX, ZFPM2, Malignant pleural mesothelioma, Diagnostic biomarker

## Abstract

**Background:**

Malignant pleural mesothelioma (MPM) is a rare and aggressive cancer that develops in the pleural and outer layer of tissues surrounding the lungs. MPM is primarily caused by occupational exposure to asbestos and results in a poor prognosis. Effective therapeutics as well as early diagnostics for the MPM are still lacking. To identify potential diagnostic biomarkers for MPM, we performed bioinformatics analysis of public database.

**Methods:**

Utilizing databases from Cancer Cell Line Encyclopedia (CCLE) and Gene Expression Omnibus (GEO), we identified several potential candidates that could act as MPM biomarkers. We carried out additional molecular analyses of these potential markers using MPM patient tissue samples via quantitative polymerase chain reaction.

**Results:**

We identified Lysyl oxidase (LOX), Lysyl oxidase homologs 1&2 (LOXL1& LOXL2) Zinc Finger Protein, FOG Family Member 2 (ZFPM2) as potential diagnostic biomarkers for MPM. In this study, we found that the LOX family and ZFPM2 showed comparable diagnostic ability to Fibulin-3 or mesothelin (MSLN) and would be better potential biomarkers than Sulfatase 1 (SULF1), Thrombospondin 2 (THBS2) and Cadherin 11 (CDH11).

**Conclusions:**

LOX family and ZPFM2 were identified as novel MPM diagnostic biomarkers which could strengthen MPM clinical diagnostic capabilities.

## Background

Malignant pleural mesothelioma (MPM) is a highly malignant tumor which occurs in the pleural mesothelial tissues covering the lung [1]. It is well known that MPM onset is usually due to asbestos exposure in an industrial environment [2]. Although there are various advanced therapeutic approaches, including surgical and chemical therapies to treat MPM, the mean overall survival ranges from six to eighteen months; and the five-year survival rate is < 16% [3–5]. Due to the prolonged latency of asbestos, asbestos mediated-MPM commonly occurs from 20 to 50 years following exposure. Based on exposure rates it is anticipated that the rate of MPM cases will increase over the next several decades [6]. Importantly, due to the high risk of MPM, use of asbestos has recently been banned in many developing countries [7, 8]. Even with considerable efforts to decrease asbestos use, further screening for potential biomarkers is necessary to aid in earlier diagnoses of the disease than is currently achievable with the available MPM biomarkers including Mesothelin, Fibulin-3 and Calretinin 2 (CALB2).

Mesothelin (MSLN) is translated into a pro-protein form, which is subsequently cleaved to into the soluble mesothelin-related protein (SMRP) and megakaryocyte potentiating factor [9, 10]. The SMRP has been detected in the serum of 84% of the MPM patients making it reasonably sensitive and suggesting its potential to act as an MPM diagnostic marker [11].

Fibulin-3 is a glycoprotein encoded by the epidermal growth factor-containing fibulin-like extracellular matrix protein-1 (EFEMP1) gene. Fibulin-3 is known to contribute to cell proliferation and/or migration. As one of the best MPM biomarkers, the sensitivity and specificity of Fibulin-3 have been reported in plasma and pleural effusions as 96 and 84%, and 95 and 93% respectively [12].. Calretinin 2 (CALB2), osteopontin (OPN), and Wilms Tumor 1 Protein (WT-1) have also suggested as potential candidates for an MPM diagnosis [13, 14].

In this study, we aimed to improve the diagnostic potential for MPM patients by establishing a multi-biomarker set comprising of seven individual biomarkers. To that end, we performed bioinformatic screenings using open databases - Cancer cell encyclopedia (CCLE) and Gene expression omnibus (GEO) and found four additional candidate genes including LOX, LOXL1, LOXL2 and ZFPM2 which show high expression levels in MPM patient tissues and cell lines when compared to their normal counterparts. We believe this multi-biomarker set could be further developed into an MPM diagnostic kit to improve the MPM diagnostic power in clinics.

## Materials and methods

### In silico analysis

Microarray data of cancer cells were obtained from the CCLE (https://portals.broadinstitute.org/ccle). Fourteen malignant pleural mesothelioma and thirty-two lung adenocarcinoma cell lines were selected and analyzed. Based on the collected data, a heat map was generated by the CCLE portal. Microarray data of human tissue was downloaded from the GEO (https://www.ncbi.nlm.nih.gov/geo) portal website. Nine normal and forty MPM tissue samples were included in the “Malignant Pleural Mesothelioma” data (accession number GSE2549) [15]. Collected biomarker candidate genes were analyzed via the following statistical methods.

### Statistical analysis

The statistical analyses were performed using Graphpad PRISM 6.0 and IBM SPSS Statistics 24 software. Receiver-operating characteristic (ROC) analyses was carried out to determine the accuracy of the biomarker candidates identified from the Malignant Pleural Mesothelioma dataset. Youden’s method was used for determination of an optimal cutoff point in the ROC curve to maximize sensitivity and specificity. To determine the statistical significance, a two-tailed unpaired t test was performed.

### Tissue collection

Human normal or MPM tissue samples were collected from bronchioalveolar lavage (BAL) fluid of non-cancerous or MPM patients. Fluid samples were centrifuged, and RNA was isolated for quantitative real-time PCR (qPCR) assays.

### Quantitative real-time PCR

Total RNA was prepared from patient samples using TRIzol reagent (Invitrogen) and reverse-transcribed into cDNA using the qPCR RT Master Mix (Toyobo). mRNA expression levels of genes of interest were determined by quantitative real-time PCR performed using an ABI Prism 7900HT Sequence Detection System (Applied Biosystems) with SYBR-green real-time PCR master mixes (Life Technologies). Data were analyzed by △△Ct method with the rRNA 18S gene used as a reference [16]. Gene-specific primers are listed in Table 1.

**Table 1:** Gene Specific Primer sequence (F: Forward, R: Reverse)

Gene	Primer sequence
18 s	**F:** 5′-ACC GCA GCT AGG AAT AAT GGA-3′
	**R:** 5′-GCC TCA GTT CCG AAA ACC A-3’
Fibulin-3	**F:** 5′-GGG AGC AGT GCG TAG ACA TAG-3’
	**R**: 5′-GCT GCC AAT TGA AAC CCA GG-3’
MSLN	**F:** 5′-GGA TGA GCT CTA CCC ACA AGG-3’
	**R:** 5′-ACT TGC GAA TGT CCT CAG GG-3’
CALB2	**F:** 5′-CTG CCT GTC CAG GAA AAC TTC-3’
	**R:** 5′-GTA GCC GCT TCT ATC CTT GTC-3’
LOX	**F:** 5′-TCT GGC CAG TAC AGC ATA CAG-3’
	**R:** 5′-CTT GGT CGG CTG GGT AAG AA-3’
LOXL1	**F:** 5′-TCT GGC CAG CAC AGC CTA T-3’
	**R:** 5′-GTT GGG GAG GAA GTC TGC TG-3’
LOXL2	**F:** 5′-ACT GCC ACA TAG GTG GTT CC-3’
	**R:** 5′-CGG GGA CAG CTG GTT GTT TA-3’
ZFPM2	**F:** 5′-TGT GTA CAG CAA AGG GGG TC-3’
	**R:** 5′-TGG CAG CTT GTA GCC TTG AG-3’
THBS2	**F:** 5′-TGA GGA CCT GGA CGA GTG TG-3’
	**R:** 5′-GCT GGT TCC CTC TGT ATC GG- 3’
SULF1	**F:** 5′-GCA GTG CAA CCC AAG ACC TA-3’
	**R:** 5′-CCA TCC CAT AAC TGT CCT CTG T-3’
CDH11	**F:** 5′- ACA AGG ATG ACA CGG CCA AT-3’
	**R:** 5′-GCC TGC TGT GTT ATC TCG GT-3’

## Results

### Genetic signatures differentiating MPM from non-MPM cells

**Fig. 1:**
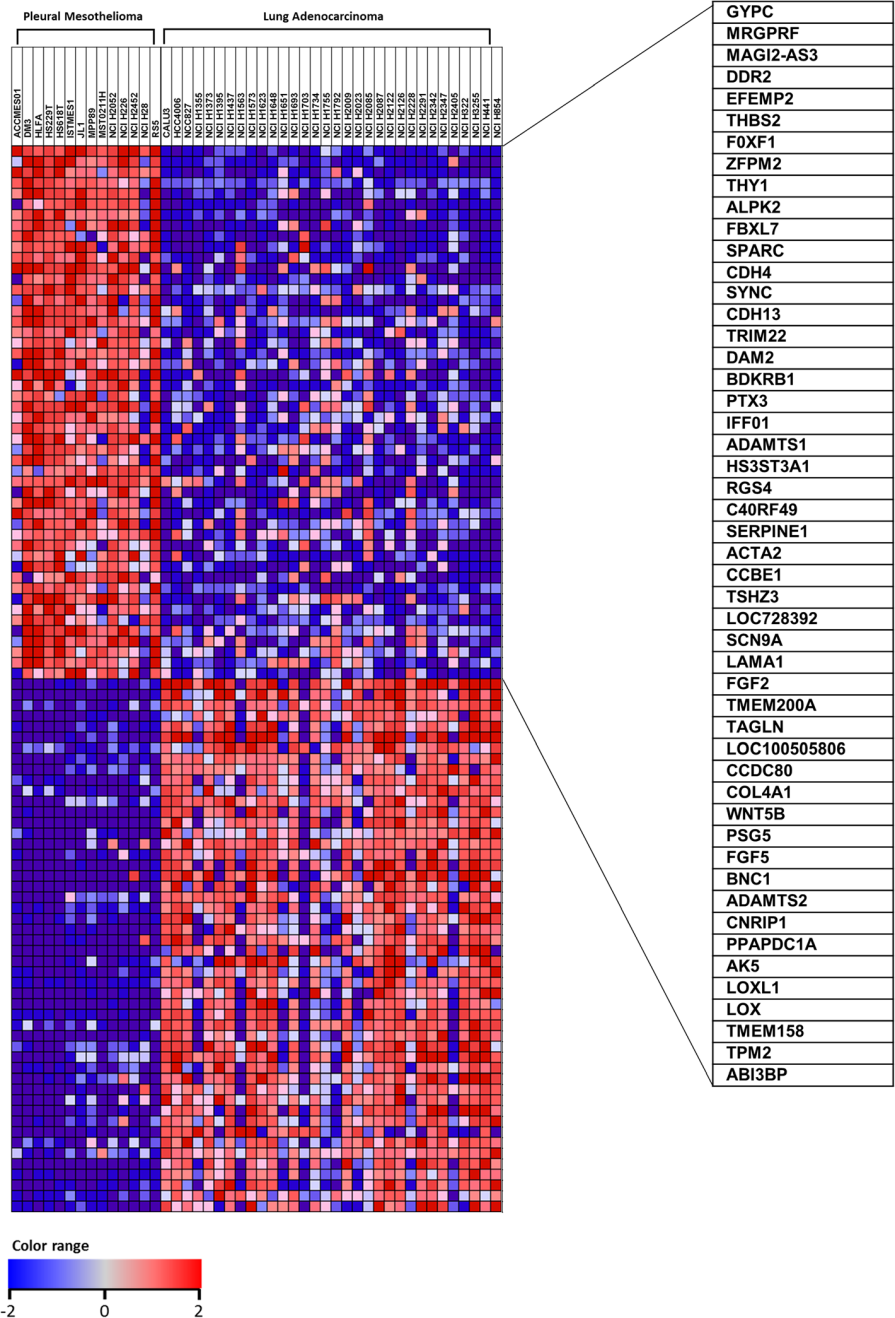
Differentiated gene expression between pleural mesothelioma and lung adenocarcinoma. From CCLE database, gene expression signature was compared between 14 MPM and 32 lung adenocarcinoma cell lines. The upper portion in the heat map listed 50 genes showing upregulated expression of mRNA in MPM cell lines. The heat map was generated based on expression score in logarithmic scale

To identify novel MPM biomarkers, we first performed bioinformatic analyses using the CCLE database which provides mRNA expression datasets for > 1100 cancer cell lines [17]. Gene expression patterns were comprehensively analyzed between 14 MPM cell lines and 32 lung adenocarcinoma cell lines using Gene Set Enrichment Analysis (GSEA). Finally, we identified a unique genetic signature which differentiates MPM from lung adenocarcinoma cell lines (Fig. 1, Additional file 1). The genetic signature includes the top 50 genes showing upregulated expression as candidate genes for potential MPM biomarkers. The genetic signature was further analyzed and confirmed as to whether it could specifically represent MPM.

### Seven genes as potential MPM biomarker candidates

**Fig. 2:**
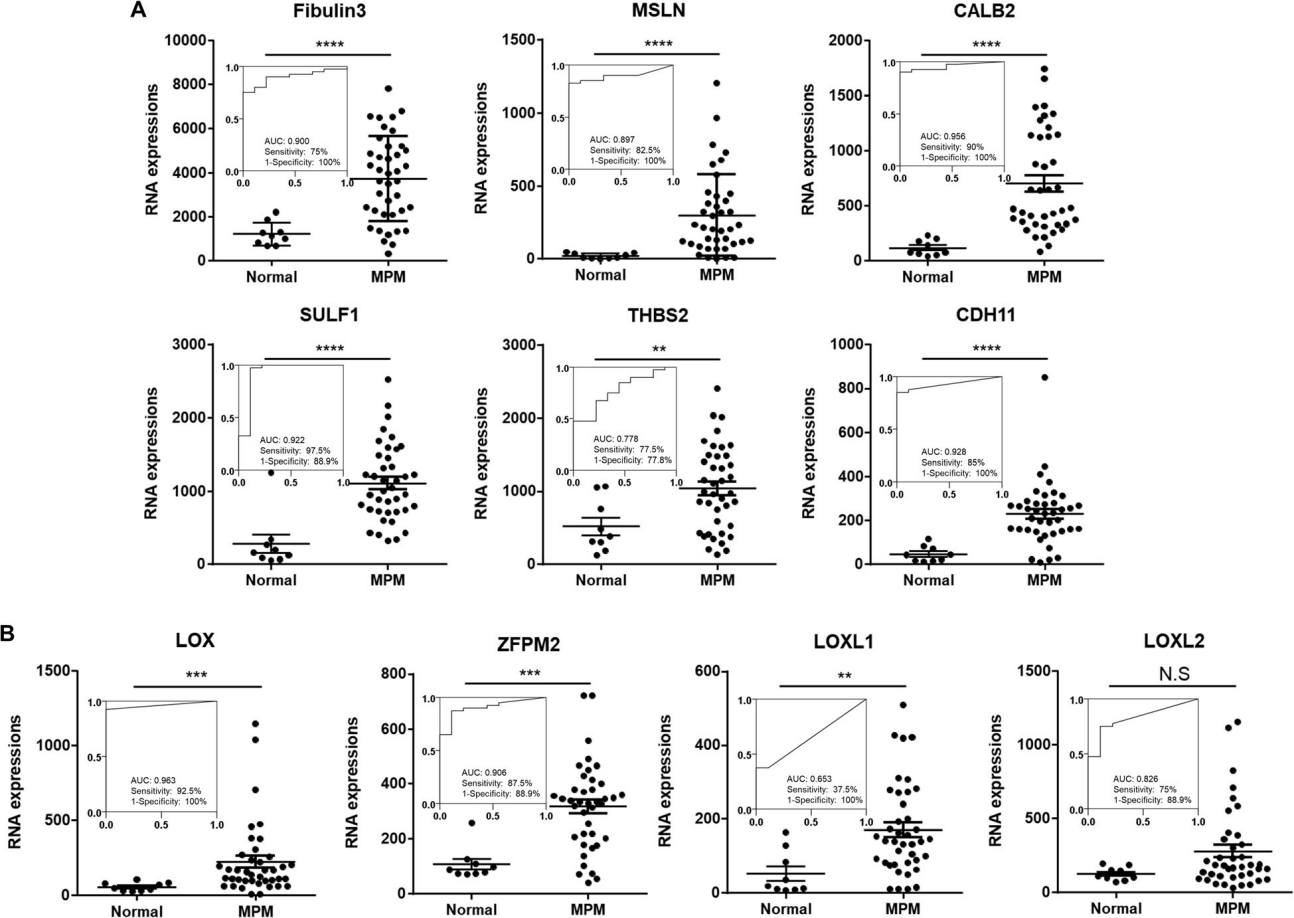
Diagnostic potential of LOX family and ZFPM2. Using the GEO database (GSE2549), mRNA expression of known biomarker (**a)** or candidate genes (**b**) were analyzed in normal (*n* = 9) and MPM tissues (*n* = 40). Each dot represents one sample, values are mean ± SEM of each groups. Statistical analysis between normal and MPM groups was executed using unpaired Student t-test. (**** *p* < 0.0001, *** *p* < 0.001, ** *p* < 0.01, * *p* < 0.05). The In-let represents ROC curves illustrating diagnostic ability of known biomarkers (**a**) and candidates (**b**). X and Y axes stands for sensitivity as well as 1-specificity, respectively. Maximal sensitivity and specificity were determined by Youden’s method. AUC (Area Under Curve)

We identified one hundred genes as potential candidates from the in silico analyses and wanted to narrow this down into a manageable number of genes. This was accomplished using multiple independent datasets for confirmation testing. We utilized the GEO database that provides microarray and functional genomic datasets from next-generation sequencing studies. A functional genomics dataset from human MPM tissues [15] was also used to validate the in silico genetic signature. These data were derived from normal pleural or lung specimens (*n* = 9) and MPM surgical specimens (*n* = 40). We first validated the reliability of the tissue transcriptomic data using the well-known MPM biomarkers Fibulin-3, MSLN and CALB2 which showed that these genes had significantly higher expression in human MPM tissues when compared with their normal counterparts (Fig. 2a). Fibulin-3 showed the highest sensitivity and specificity in the ROC curve analysis (Fig. 2a). Using the same approach as above, we analyzed the expression of the one-hundred potential MPM candidate genes identified from the GEO dataset (Fig. 1). Interestingly, we found that LOX, LOXL1, LOXL2, ZFPM2, SULF1, THBS2, and CDH11 showed significantly higher expression in the MPM tissues compared to the normal samples (Fig. 2a and b). It is important to reiterate that three of these genes (SULF1, THBS2 and CDH11) have been suggested as MPM biomarkers in previous studies [18]. More significantly, the ROC curve analysis revealed that the LOX family and ZFPM2 showed a high sensitivity and specificity to act as MPM biomarkers (Fig. 2b). Taken together, these data suggest that the seven candidate genes could serve as potential novel MPM biomarkers.

### Evaluating the potential of LOX family and ZFPM2 as MPM biomarker candidates in MPM tissues

**Fig. 3:**
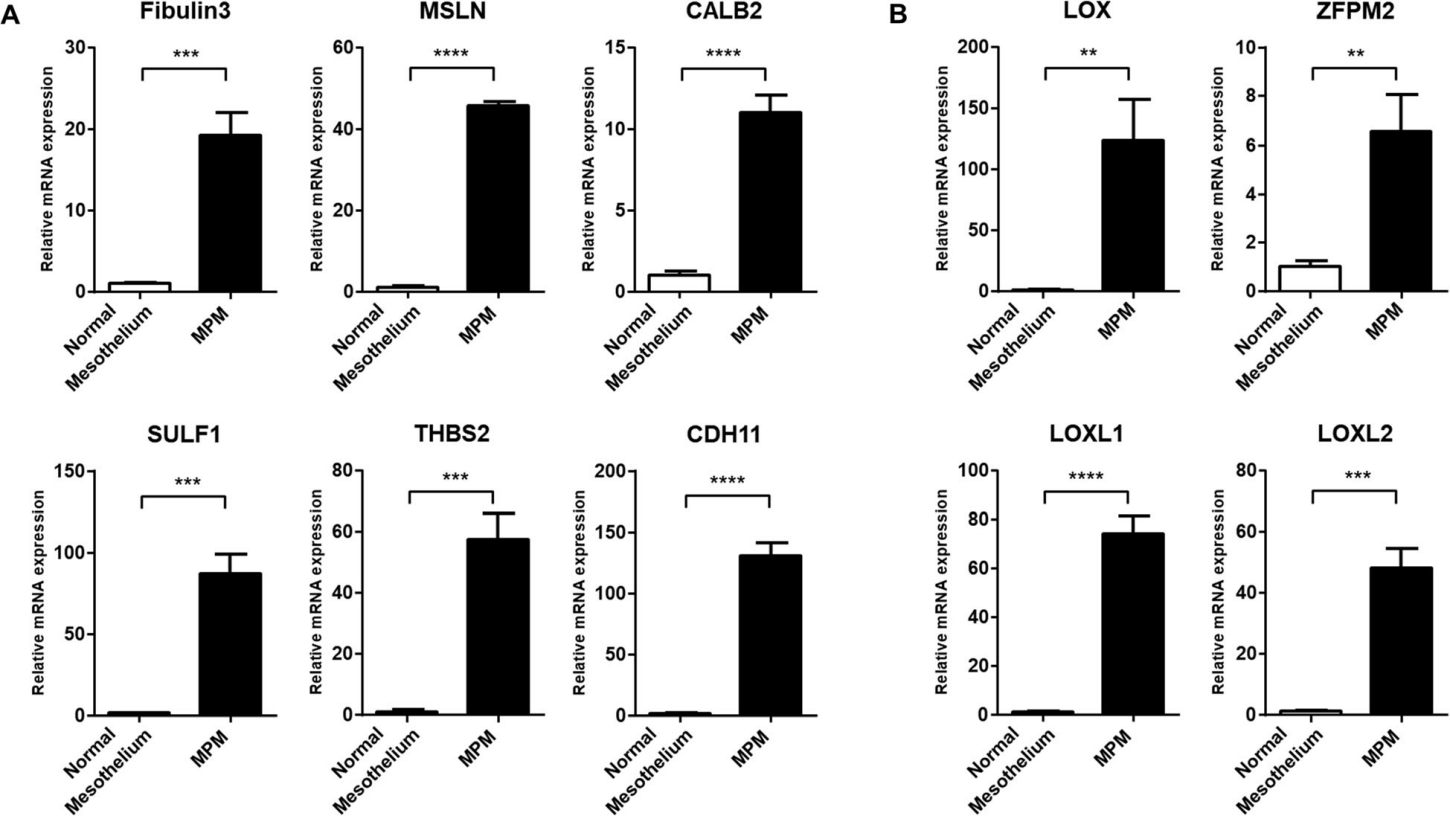
mRNA expression of the candidate biomarkers in patient samples. Using the qPCR assay, mRNA expression was surveyed for the known (**a**) and the candidate MPM biomarkers (**b**) in mesothelium tissues isolated from a non-cancer patient and a MPM patient. Values are mean ± SEM of each groups. The Statistic between normal and MPM groups was analyzed using the unpaired Student t-test. (**** *p* < 0.0001, *** *p* < 0.001, ** *p* < 0.01, * *p* < 0.05)

As proposed, a subset of the biomarker candidates for MPM diagnostics was further investigated to determine if their expression signatures could be validated in novel set of clinically confirmed samples. Pleural effusion samples from non-cancer patients and MPM patients were obtained under Institutional Review Board (IRB) approval. Using the qPCR assay, we profiled the mRNA expression of the candidate genes in the tissue samples. Consistent with the data from the GEO analysis, expression of the known MPM biomarkers were significantly higher in the MPM tissue when compared with the normal tissue (Fig. 3a). Following the validation of the known biomarkers, we also checked the mRNA expression levels for the four new proposed MPM biomarker candidates in the same patient samples. Similarly, the expression of the four MPM biomarker candidates LOX, LOXL1, LOXL2 and ZFPM2 were shown to be significantly increased in the MPM tissues when compared to the non-cancer patient samples (Fig. 3b). Taken together, these data suggest that these candidates could be included as members of a panel, together with previously known biomarkers, for improved MPM diagnoses.

## Discussion

Early detection and treatment of cancer using specific biomarkers helps to reduce disease progression. Thus, multi-biomarker sets rather than a single gene biomarker could increase the diagnostic power and provide patients with a more sensitive diagnostic for mesothelioma [19–24]. In this present study, we utilized the CCLE and GEO databases for a comparative analyses of gene expression patterns between MPM patients and controls. Based on our bioinformatic analyses, we were able to identify a subset of genes which show a higher expression levels in the MPM group when compared to the normal or lung adenocarcinoma group.

The diagnostic potential of the gene candidates was further analyzed together with three previously well-known MPM biomarkers Fibulin-3, MSLN, and CALB2. In particular, we noticed that the diagnostic power of LOX family was comparable to or even greater than that of Fibulin-3 which has shown the most significant diagnostic potential in the literature [12]. We further confirmed the specificity of the MPM biomarkers in the paired set of a normal mesothelium and patient fluid sample. The assembled biomarker panel consisting of seven genes showed significantly increased sensitivity for diagnosing MPM from pleural fluid samples.

One notable finding in this study was that we identified several LOX isoforms as potential MPM biomarker candidates. The LOX gene encodes lysyl oxidase that induces crosslinking of elastin and collagen by catalyzing oxidative deamination on lysine or hydroxylysine residues, and the other LOXL family members do as well. This biochemical function is known to be important in maintaining organismal structure [25–27]. Therefore, dysfunction of LOX and the LOXL family contributes to various types of diseases including liver fibrosis, cardiovascular disease, and cancer. In cancer biology, hypoxia-inducible factor induces LOX expression, which contributes to hypoxic metastasis in breast cancer [28] and is also associated with a poor prognosis and invasion in lung cancer [29]. However, little is known about LOX and LOXL family involvement in mesothelioma. One might think that the MPM specific expression pattern of the LOX and LOXL family needs to be further studied in the MPM cancer biology.

The ZFPM2 gene encodes the Friend of GATA-2 (FOG-2) protein which is a transcription cofactor interacting with GATA family members transcriptionally involved in diverse biological functions including cardiac, pulmonary, gonadal development and hematopoiesis [30–36]. Similarly, we found that ZFPM2 has a high diagnostic potential from the database analyses and this was confirmed via performance of a case-control study.

## Conclusions

Taken together, the four biomarker candidates (LOX, LOXL1, LOXL2, ZFPM2) identified in the current study could be combined with the previously proposed biomarkers to strengthen the diagnostic power for MPM patients.

## Additional file

**Additional file 1.** Gene expression list in pleural mesothelioma compared to lung adenocarcinoma. Note that expression logarithmic score was presented to each gene list by comparing 14 MPM and 32 lung adenocarcinoma cell lines from CCLE database.

## Data Availability

All of data and materials are available from the corresponding author upon reasonable ask.

## Abbreviations

BAL: Bronchioalveolar lavage
CALB2: Calretinin 2
CCLE: Cancer cell line encyclopedia
CDH11: Cadherin 11
FOG-2: Friend of GATA-2
GEO: Gene expression omnibus
GSEA: Gene set enrichment analysis
IRB: Institutional review board
LOX: Lysyl oxidase
MPM: Malignant pleural mesothelioma
MSLN: Mesothelin
OPN: Osteopontin
ROC: Receiver-operating characteristic
SULF1: Sulfatase 1
THBS2: Thrombospondin 2
WT-1: Wilms tumor 1 protein
ZFPM2: Zinc finger protein, fog family member 2
